# Evaluation of the level of cortisol, capillary blood glucose, and blood pressure in response to anxiety of patients rehabilitated with complete dentures

**DOI:** 10.1186/s12903-019-0763-z

**Published:** 2019-05-03

**Authors:** Marcelo Coelho Goiato, Emily Vivianne Freitas da Silva, Nádia Biage Cândido, Adhara Smith Nóbrega, Rodrigo Antonio de Medeiros, Doris Hissako Sumida, Fernando Yamamoto Chiba, Daniela Micheline dos Santos

**Affiliations:** 1 Department of Dental Materials and Prosthodontics, Aracatuba Dental School, Sao Paulo State University (UNESP), Jose Bonifacio St., 1153, Vila Mendonca, Aracatuba, Sao Paulo 16015-050 Brazil; 2 Department of Dental Materials and Prosthodontics, UNIEURO University Center, Brasilia, Federal District Brazil; 3 Department of Basic Sciences, Aracatuba Dental School, Sao Paulo State University (UNESP), Aracatuba, Sao Paulo Brazil; 4 Department of Preventive and Social Dentistry, Aracatuba Dental School, Sao Paulo State University (UNESP), Aracatuba, Sao Paulo Brazil

**Keywords:** Complete denture, Anxiety, Blood glucose, Blood pressure

## Abstract

**Background:**

The aim was to analyze the levels of stress of edentulous patients through the state-trait anxiety inventory (STAI) and salivary flow through the visual analogue scale (VAS) xerostomia questionnaire, as well as analyze the levels of cortisol, capillary blood glucose, and blood pressure (BP) before and after the installation of complete dentures.

**Methods:**

Fifty patients were evaluated. The STAI and VAS xerostomia questionnaire were applied before the installation of the prosthesis, on the day of its installation, and 1 month after the last recall visit. The BP measurement, as well as salivary and blood collections, were performed before the installation of the prothesis, and 1 month after the last recall visit. Data from the VAS xerostomia questionnaire and cortisol levels were submitted to ANOVA and the Tukey test (*P* = .05). Data from the STAI, as well as blood glucose and BP levels, were submitted to the Chi-square test (*P* = .05). The correlation between cortisol and blood glucose and between cortisol levels and BP was analyzed.

**Results:**

There was no statistically significant association between the questions of the VAS xerostomia questionnaire, STAI-state and STAI-trait scores, or the periods analyzed. However, the cortisol level collected in the morning decreased after the installation of the prosthesis. There was a correlation between cortisol and blood glucose and BP levels.

**Conclusions:**

The installation of complete dentures was beneficial for patients since it was probably responsible for the cortisol level reduction.

## Background

Edentulous patients have difficulty feeding, chewing, and impaired phonetics. In addition, dental losses lead to a reduction of bone tissue and muscle tone with an unfavorable effect on facial esthetics [1–3]. As a result, these factors can lead to emotional alterations and a reduction in the quality of life of these patients, raising their stress levels [4, 5].

Stressful situations can trigger cardiovascular responses [6], which actively participate in alertness adaptations and are subject to neurohumoral influences. These responses mainly result in increased heart rate and contractility, an increase in blood pressure (BP) [7], and higher activity of the hypothalamic axis [6], and are linked with the activation of immune cells and secretion of several hormones [8], especially cortisol, which is considered a stress hormone [9].

Cortisol acts on two distinct fronts that results in increased amounts of glucose in the bloodstream. It stimulates gluconeogenesis in the liver, and the glucose produced is released into the bloodstream and stored as glycogen [10]. In addition, by potentiating the effects of epinephrine [11], it elevates glycogenolysis in the liver, thus releasing a large amount of glucose into the bloodstream within minutes [12]. Excess gluconeogenesis and glycogenolysis cause hyperglycemia, which may favor episodes of diabetes. It is interesting to note that disorders of glucose metabolism can cause complications such as cardiovascular diseases, including systemic arterial hypertension (SAH), coronary artery disease, and heart failure [13].

It is essential to evaluate, from functional and esthetic points of view, the relationship and importance of dental rehabilitation with the general health of the edentulous patient, by checking the level of stress through hormonal markers and their systemic health. The aim of this study was to analyze the levels of stress of edentulous patients through the state-trait anxiety inventory (STAI) and salivary flow through the visual analogue scale (VAS) xerostomia questionnaire, as well as analyze the levels of cortisol, capillary blood glucose, and blood pressure (BP) before and after the installation of complete dentures, verifying the correlation between the salivary cortisol level and the blood glucose and BP levels.

This study presents the null hypothesis that the rehabilitation with complete dentures would not influence (1) the salivary cortisol level, (2) anxiety and salivary flow, and (3) capillary blood glucose and BP.

## Methods

The present study was approved by the Research Ethics Committee of the Aracatuba Dental School – UNESP (Protocol N° 085011/2014). The sample size was defined considering a 0.05 level of significance, 80% power, and a medium effect size. The results of the calculation showed that 28 patients were required for the study. Edentulous patients using complete dentures for at least 10 years, who needed replacement of the complete dentures, were evaluated through anamnesis and clinical examination. Fifty patients were selected based on the inclusion and exclusion criteria.

The inclusion criteria were total bimaxillary edentulism, presence of adequate support tissue health with a mucosa with medium resilience, and proper capacity to understand and answer the questionnaire and expel the saliva in the appropriate container. The exclusion criteria were use of a cortisol-based drug, recent emotional stress, smoking habits, pain after the prosthesis installation, and presence of infection. New prostheses were made for the patients by the same professional, following the procedures recommended by Zarb et al. [14]. Patients received instructions about the use and hygiene of the new dentures.

The STAI and VAS xerostomia questionnaire were applied before the prosthesis insertion (initial), on the day of its installation, and 1 month after the last recall visit (final). The VAS xerostomia questionnaire allowed subjective evaluations of the salivary flow through questions related to xerostomia, which were answered by the patients themselves by marking a vertical trace on a horizontal scale of 0 to 100 mm [15]. Proposed by Pai et al. [15], the questionnaire evaluates two main aspects of the salivary flow: dryness of the oral mucosa (lips, mouth, tongue, or throat) and oral functions impaired by the sensation of dry mouth (difficulty swallowing or speaking). Two global items regarding dryness of the mouth are analyzed: salivary amount and thirst sensation.

The STAI is a self-evaluation questionnaire divided into two parts: assessment of state-anxiety and trait-anxiety. Each of these parts is composed of 20 statements, with answers given on a scale ranging from 1 to 4, where state means how the subject feels “at the moment” and trait indicates how they feel generally. The score of each part varies from 20 to 80 points, indicating low (20–30), medium (31–49), and high (greater than or equal to 50) degrees of anxiety [16, 17].

Six BP measurements and salivary and blood collections were performed: three measurements were performed before the prosthesis installation (initial) and three measurements were performed one month after the last recall visit (final), in order to guarantee the absence of pain and good adaptation of the patient to the prosthesis. The aim of the collections was to verify if there was a correlation between the cortisol levels and blood glucose and between the cortisol levels and BP. For the collections and measurements, visits were previously scheduled and performed at the home of the volunteer patients three-times a day: morning (after fasting for approximately 12 h); afternoon (2 h postprandial) and; evening (2 h postprandial) [18]. The team wore caps, masks, gloves, and lab coats for the collections and measurements. The lab coat was green, aiming to avoid white coat syndrome, characterized by BP peaks when the patient is approached by a professional dressed in a white coat [19].

Patients were advised not to eat or brush their teeth within 2 h before the saliva collection to avoid contamination from food and dentifrice residues [20]. The oral cavity was previously cleaned with a filtered-water rinse. To stimulate salivation, the patient was instructed to gently chew Parafilm (0.29 g, Parafilm ‘M’; American National Can TM), which consists of a flexible, odorless, disposable film that does not absorb saliva, for 5 min [21]. After this period, the Parafilm and saliva were removed from the mouth and placed in the Salivette tube (Salivette(r); Genese Produtos Diagnosticos Ltda). Every salivary sample containing blood was discarded. After collection, all samples were stored in ice in a polystyrene box for 20 min, in order to standardize the refrigeration time before freezing. Samples were then stored at − 20 °C until centrifugation, which was performed at 2000 rpm for 5 min, after which the supernatant was separated and stored at − 20 °C [20, 22]. The salivary cortisol concentration was established in duplicate by the competitive enzyme linked immunoabsorbent assay (ELISA) method. The Diametra DKO020 commercial kit was used, according to the manufacturer’s recommendations [20].

In order to verify blood glucose levels, antisepsis of the third finger digital pulp of each patient was performed using a 70% alcohol-soaked cotton ball, and a blood sample (one drop) was obtained using a disposable lancet (Accu-Chek Softclix Pro; Roche) [23, 24]. The blood glucose was measured using a specific monitor (Accu-Chek Performa, Roche), and a blood glucose level between 70 and 99 mg/dl was considered normal; between 100 and 125 mg/dl, prediabetes (glucose intolerant); and above 125 mg/dl, diabetes, according to the American Diabetes Association (ADA) criteria [25].

A stethoscope and sphygmomanometer were used for BP measurement and the patient was seated with the arm resting at the level of the heart [26]. The values obtained were classified according to the American Heart Association: < 120/< 80 mmHg considered normal; 120–139/80–89 mmHg, prehypertension; 140–159/90–99 mmHg, hypertension stage 1 (mild); 160–179/100–109, hypertension stage 2 (moderate) and; > 179/> 109, hypertensive crisis (severe) [27].

Statistical analysis was performed using SPSS software version 21.0 (Statistical Package for the Social Sciences, IBM Corp). Data from the VAS xerostomia questionnaire were submitted to one-way analysis of variance (ANOVA) and the Tukey test (*P* = .05). Data from the STAI, blood glucose levels, and BP levels were submitted to descriptive statistics and the Chi-square test (*P* = .05). Data from salivary cortisol levels were submitted to two-way repeated-measures ANOVA and the Tukey test (*P* = .05). The Spearman correlation between cortisol levels and blood glucose and between cortisol levels and BP were performed.

## Results

Of the 50 patients selected for the study, two of them moved from the city before the conclusion of treatment and seven did not want to participate after the first application of questionnaires, BP measurement, and salivary and blood collection. A total of 41 patients (15 males and 26 females) were included in the study. The mean age of the patients was 73.7 ± 9.30 years, except for 5 patients who did not report this data. All patients were retired, lived with their families, and had no diet restrictions. There was no significant statistical difference (*P >* .05) among the periods analyzed for each question of the VAS xerostomia questionnaire (Table 1). However, there was a progressive increase in the mean values over time for questions 1 (difficulty in speaking due to dryness), 2 (difficulty in swallowing due to dryness), 3 (how much saliva is in the mouth), and 6 (dryness of the lips). For all other questions, there was an increase in the value between the initial analysis and the day of the prosthesis installation, with a reduction 1 month after the end of treatment.

**Table 1:** Mean values ± standard deviation for questions of VAS xerostomia questionnaire

Questions of VAS xerostomia questionnaire	Periods analyzed			
	Initial	Installation	Final	*P*
Question 1	6.05 ± 4.18	6.58 ± 3.83	6.77 ± 3.66	0.686
Question 2	6.45 ± 3.89	7.13 ± 3.49	7.18 ± 3.39	0.590
Question 3	3.29 ± 3.43	3.65 ± 3.02	3.82 ± 3.34	0.760
Question 4	4.23 ± 3.26	4.58 ± 2.82	4.22 ± 3.06	0.828
Question 5	3.46 ± 3.09	4.17 ± 2.69	3.95 ± 2.72	0.516
Question 6	4.65 ± 2.98	4.99 ± 2.66	5.01 ± 3.02	0.815
Question 7	3.89 ± 2.79	4.50 ± 2.56	4.49 ± 3.04	0.532
Question 8	5.88 ± 3.71	6.77 ± 2.99	6.48 ± 3.23	0.468

It was verified that the low degree of state-anxiety was the most frequently observed in all periods analyzed (Table 2). There was no statistically significant association between degrees of state-anxiety and periods of analysis (*P =* .175).

**Table 2:** Distribution of frequency (and percentage) of state-anxiety degrees, according to different periods analyzed (initial, on day of prosthesis installation, and final)

Periods analyzed	State-anxiety degree		
	Low	Medium	High
Initial	38 (92.7%)	3 (7.3%)	0
Installation	34 (82.9%)	7 (17.1%)	0
Final	32 (78.0%)	9 (22%)	0
Total	104	19	0

For the trait-anxiety, the medium degree was the most frequently observed in all periods analyzed (Table 3). There was no statistically significant association between degrees of trait-anxiety and periods of analysis (*P* = .472).

**Table 3:** Distribution of frequency (and percentage) of trait-anxiety degrees, according to different periods analyzed (initial, on day of prosthesis installation, and final)

Periods analyzed	Trait-anxiety degree		
	Low	Medium	High
Initial	16 (39.0%)	24 (58.6%)	1 (2.4%)
Installation	9 (19.5%)	29 (73.2%)	3 (7.3%)
Final	14 (34.1%)	25 (61.0%)	2 (4.9%)
Total	39	78	6

An interaction between the time of day and period analyzed influenced the salivary cortisol level (*P* < .001). According to Table 4, regardless of the periods analyzed (initial and final), higher levels of cortisol were observed in the saliva collected in the morning, with statistical difference. In addition, when comparing the different periods analyzed, the initial cortisol was statistically higher than the final cortisol only in the quantification performed in the saliva collected in the morning period.

**Table 4:** Mean values ± standard deviation of salivary cortisol level for different times of day (morning, afternoon, evening) at initial and final periods of analysis

Period analyzed	Time of day		
	Morning	Afternoon	Evening
Initial	16.84 (14.19) Aa	6.30 (8.85) Ab	2.22 (2.14) Ab
Final	9.35 (8.30) Ba	5.23 (6.47) Ab	2.88 (5.60) Ab

Different uppercase letters in column and lowercase letters in row indicate statistically significant difference by Tukey test (*P* < 0.05)

**Fig. 1:**
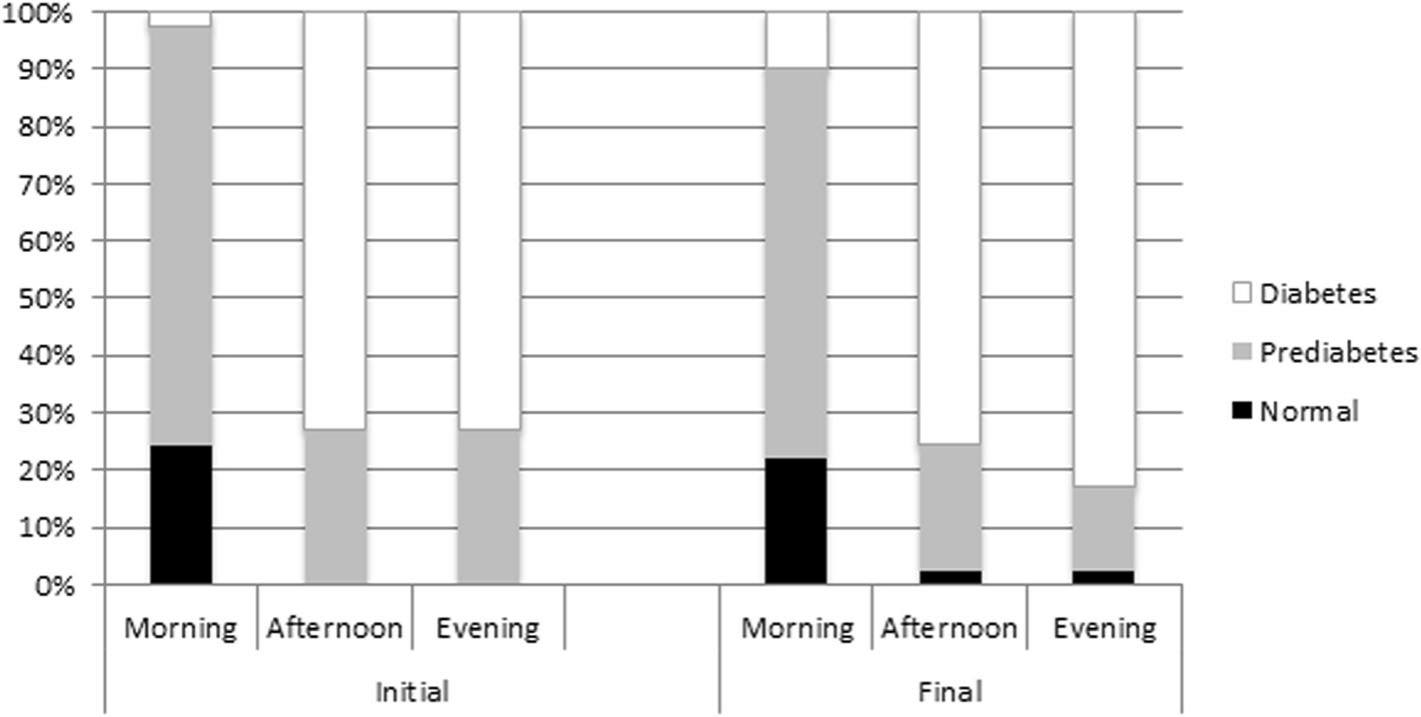
Percentage distribution of blood glucose levels according to time of day at initial and final periods of analysis

Regarding the blood glucose levels, the prevalence of the diabetic level was lower in the morning, regardless of the periods analyzed. However, its prevalence increased for other times of the day, with a higher percentage in the final period of analysis (Fig. 1). There was a statistically significant association (*P* < .001) between the blood glucose levels according to times of day and periods of analysis.

There was a significant and negative correlation (correlation of −.405) between the cortisol level and the blood glucose level of the patients evaluated.

**Fig. 2:**
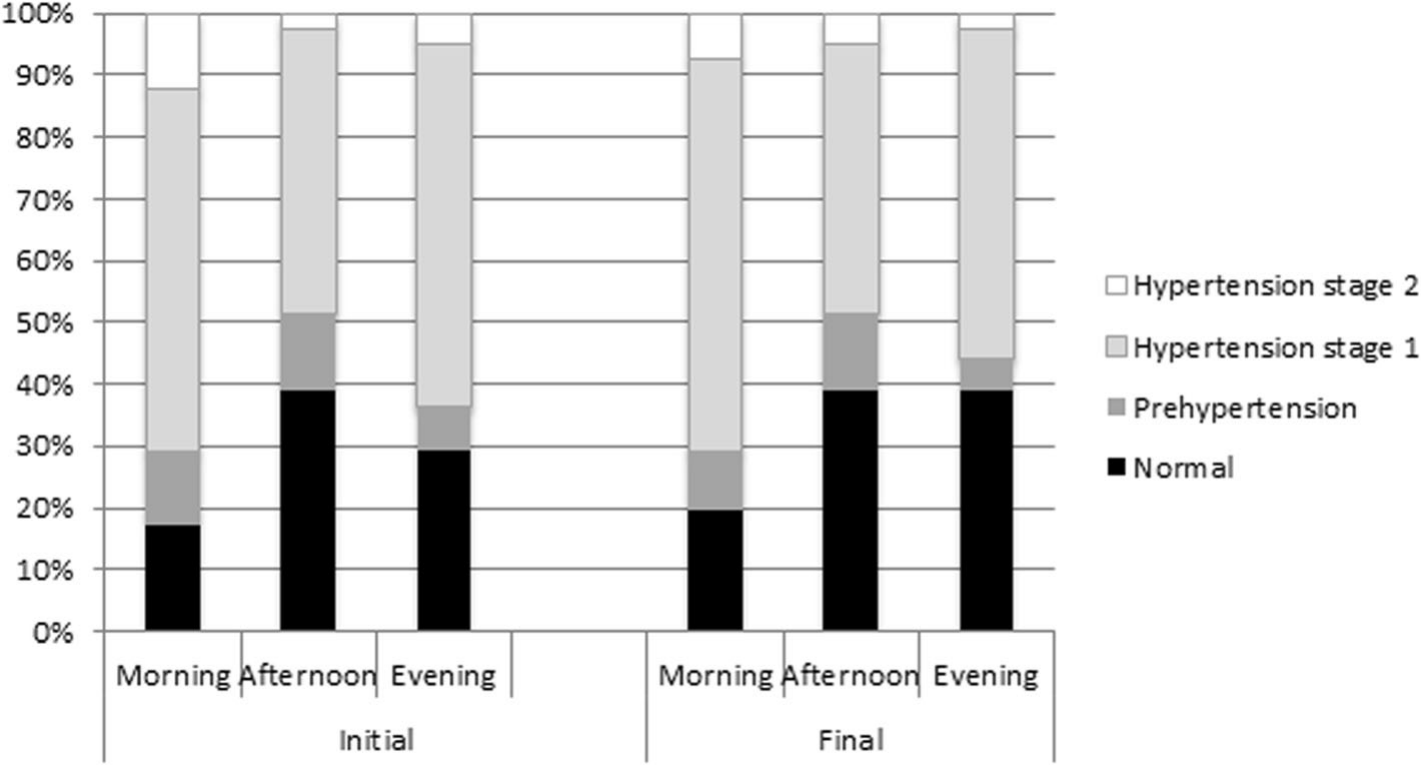
Percentage distribution of BP levels according to time of day at initial and final periods of analysis

When analyzing the BP results, it was verified that, regardless of the period analyzed and time of the day, hypertension stage 1 level was the most frequent in the patients studied. Comparing the initial and final periods, an increase in the normal BP frequency and a reduction in the frequency of the other levels was observed (Fig. 2). No statistically significant association was found in the individual analysis between the BP levels, according to times of day and initial (*P* = .231) and final periods (*P* = .328).

There was a significant and positive correlation (correlation of .193) between the cortisol levels and the BP levels of the patients evaluated.

## Discussion

The null hypothesis that the rehabilitation with complete dentures would not influence the salivary cortisol level was rejected, since the cortisol from the saliva collected in the morning decreased after the prosthesis installation. Regarding the anxiety level and salivary flow, the null hypothesis was accepted since there was no statistically significant association between the questions of the VAS xerostomia questionnaire, STAI-state score, STAI-trait score, or the periods analyzed. The null hypothesis that the rehabilitation would not influence capillary blood glucose and BP was rejected since both had alterations after the prosthesis installation.

The VAS xerostomia questionnaire evaluates the subjective level of salivation by means of a visual analogue scale, using eight questions related to the dryness of the oral mucosa and to oral functions impaired by the sensation of oral dryness [15]. In the present study, no significant statistical difference was found among the periods analyzed for all questions evaluated. However, there was an increase in the score after the installation, when compared to the initial analysis, indicating a higher sensation of xerostomia by patients (Table 1).

It is important to emphasize that this is a subjective evaluation questionnaire, which does not necessarily represent the actual situation of patient salivation, but the perception of it. It is known that complete dentures are responsible for improving mastication, esthetics, speech and, consequently, self-esteem of the patient [28]. Some generated anxiety can be related to the result that will be obtained at the moment of the prosthesis installation, causing a sensation of salivary flow reduction, which would explain the results found.

Naumova et al. [29] evaluated the relationship between stress and level of salivary secretion and found no relation between them, since stress was not able to reduce salivary flow. However, when evaluating proteins present in the saliva, they observed an increase in their concentration after exposure to stress, concluding that the main cause of dry mouth sensation in stressful situations is not the reduction of salivary flow, but a change in the composition of the saliva.

For questions 4, 5, 7, and 8, unlike the other questions, a reduction in the sensation of mouth dryness was observed in the analysis performed 1 month after the end of treatment (Table 1). According to Wolff et al. [30], which evaluated the salivation of patients after installation of complete dentures, increased salivation may occur due to chronic stimulation of the mechanoreceptors located below the base of the dentures, which would increase the salivary flow through the pressure caused by them.

In the present study, no statistically significant association between the anxiety levels, either state- or trait-anxiety, and the periods analyzed was found (Table 2). However, the most frequent degree of state-anxiety observed was the low degree, and for the trait-anxiety it was the medium degree. The state-anxiety verifies the degree of anxiety at the precise moment the test is applied [31], and it refers to the moment when the dental care is given in this case. On the other hand, the trait-anxiety is related to the degree of anxiety in daily social and family life.

Enkling et al. [32] reported that a large number of patients associate dental appointments with high levels of anxiety, with some reporting phobia. Naumova et al. [33] grouped the causes of anxiety in patients, and pain related to dental care was the main cause of this problem. The results of the present study showed that patients attended at the clinic had a lower degree of anxiety than in their daily life, probably because the dental care of edentulous patients is not related to invasive and painful procedures. In addition, during the visits where the application of questionnaires and collection of saliva were performed, the need of these patients, who often use dental appointments as a moment of emotional and psychological support, was easily noticeable, which justifies the low degree of anxiety found.

Regarding the cortisol levels, an interaction between the time of day and period analyzed influenced their values. It is known that cortisol presents a circadian cycle, being secreted with a strong diurnal rhythm. The secretion usually has peaks in the morning, decreasing at night. Half of the total daily cortisol is secreted before dawn [9, 10]. This is the reason why cortisol levels in the morning were higher when compared to the afternoon and evening periods at the initial and final periods analyzed.

The level of secreted cortisol can be measured by assessing urine, saliva, or blood plasma [18]. The measurement technique of salivary cortisol is considered the gold standard since it can be used for the evaluation of cortisol regulation in all conditions [34]. This measurement is accessible, fast, non-invasive, and effective since it does not affect the results due to the patient’s stress on needle contact. In addition, the material collection can be performed in domicile [35] and the repetition of collections can occur in a short interval of time [34]. A positive correlation is found between blood and salivary cortisol levels [35]. Salivary cortisol samples are stable at room temperature for a week, and can be transported to the laboratory by mail or by carrier without any loss of hormone activity [36]. For these reasons, it was decided to perform the analysis of cortisol through saliva in the present study.

In the present study, the initial cortisol level was statistically higher than the final level in the saliva collected during the morning (Table 4). Old, unsatisfactorily adapted, inadequate esthetic prostheses, or the ones that cause injuries and affect the mucosa, alter the patient’s self-esteem, causing an increase in stress and in cortisol levels [37, 38]. The results found suggested that the installation of a new complete denture, after all necessary adjustments and patient adaptation, may have promoted a decrease in salivary cortisol levels.

The selected patients declared that they were neither diabetic nor hypertensive. However, some presented high glucose levels observed in diabetic patients (Fig. 1) and high BP, indicating hypertension (Fig. 2). Despite the two-hour eating and brushing restriction orientation they received, the patient was at home, without the control of the professional. Thus, it is possible that some patients did not respect the combined feeding schedule for correct glycemic analysis, especially in the final period of analysis. However, in cases of extremely high blood glucose and/or BP in all times of the day, the patient in question was advised to seek a doctor for evaluation.

Diabetes mellitus is a metabolic disorder characterized by hyperglycemia due to defects of insulin secretion or action [14]. There are two types of this disease: type 1, caused by the lack of insulin secretion, and type 2, influenced by insulin resistance. Cortisol activates the body’s response to emergency situations, increasing blood glucose and, consequently, BP, providing muscular energy [7, 9]. In the present study, there was no correlation between the salivary cortisol levels and the blood glucose or the BP levels.

The present study has some limitations, such as the impossibility of controlling all variables that may interfere with results; the absence of a control group, such as patients who did not receive new dentures [39]; and a short period of patient analysis. However, the initial condition served as a control of the rehabilitation. In addition, the reduction of cortisol levels, which probably happened due to the installation of new complete dentures, is a great benefit to the health of the patients. Therefore, the dentist should advise the patient about the periodicity of the prosthesis replacement, and must be attentive at follow-up visits to ensure adaptation to the prosthesis and absence of intraoral lesions [38], in order to maintain the low level of anxiety/stress, benefiting the patient’s health.

### Practical implications

The process of adaptation to new dentures is improved by the professional’s knowledge of the relationship between dental rehabilitation and the general health of edentulous patients.

## Conclusions

- No alteration in patient anxiety or xerostomia levels was verified through the questionnaires applied after the installation of complete dentures;
- The installation of complete dentures was beneficial for patients, since it was probably responsible for the cortisol level reduction of the saliva collected in the morning, after prosthesis adjustments and adaptation.
- There was a correlation between the salivary cortisol levels and the blood glucose levels and the BP levels.

## Abbreviations

ADA: American Diabetes Association
ANOVA: Analysis of variance
BP: Blood pressure
ELISA: Enzyme linked immunoabsorbent assay
SAH: Systemic arterial hypertension
STAI: State-trait anxiety inventory
VAS: Visual analogue scale
